# A Story of Theodore Parker

**Published:** 1892-01

**Authors:** 


### A STORY OF THEODORE PARKER.

A story of Theodore Parker's early life, which is said to be authentic, and was related, indeed, by Mr. Parker himself, was that when a boy about twelve years of age, he was at work one day on his father's farm near Lexington, and suddenly a venerable man stood by him. His silvery hair and flowing beard impressed the lad as somewhat unusual, and for some time the aged man walked along by him, talking to him earnestly of all that it was possible for a boy to become and to do in the world. It made upon the boy a lasting impression, and he repeatedly affirmed that the hour became to him a conscious date in life, one that stimulated all his latent force and aspiration. On inquiring as to whence the stranger came, no one could tell. It was a country neighborhood, where any visitor attracted attention, and as no one but the lad had seen him, he came in after years to believe that his visitor was of supernatural origin.

